# PPARγ inhibition boosts efficacy of PD-L1 Checkpoint Blockade Immunotherapy against Murine Melanoma in a sexually dimorphic manner

**DOI:** 10.7150/ijbs.42966

**Published:** 2020-03-05

**Authors:** Bogang Wu, Xiujie Sun, Bin Yuan, Fei Ge, Harshita B. Gupta, Huai-Chin Chiang, Jingwei Li, Yanfen Hu, Tyler J. Curiel, Rong Li

**Affiliations:** 1 Department of Biochemistry and Molecular Medicine, School of Medicine and Health Sciences, The George Washington University, Washington, DC 20037, USA.; 2 Department of Molecular Medicine, University of Texas Health San Antonio, San Antonio, TX 78229, USA.; 3 Department of Medicine, University of Texas Health San Antonio, San Antonio, TX 78229, USA.

**Keywords:** Melanoma, immunotherapy, PD-L1, PPARγ, obesity, sexual dimorphism

## Abstract

Immune checkpoint blockade-based immunotherapy has become standard of care for multiple cancer types. However, the overall response rates among various cancer types still remain unsatisfactory. There is a pressing clinical need to identify combination therapies to improve efficacy of anticancer immunotherapy. We previously showed that pharmacologic inhibition of PPARγ by GW9662 boosts αPD-L1 and αPD-1 antibody efficacy in treating murine mammary tumors. In addition, we defined sexually dimorphic αPD-L1 efficacy in B16 melanoma. Here, we show a sexually dimorphic response to the combination of GW9662 and αPD-L1 immunotherapy in B16 melanoma. Combination effects were observed in female, but not male hosts. Neither female oöphorectomy impairs, nor does male castration rescue the combination effects, suggesting a sex hormone-independent response to this combination therapy. In diet-induced obese females, melanoma growth remained responsive to the combination treatment, albeit less robustly than lean females. These findings are informative for future design and application of immunotherapy-related combination therapy for treating human melanoma patients by taking gender and obesity status into consideration.

## Introduction

Malignant melanoma, an aggressive type of skin cancer, has higher metastatic potential and higher mortality rate compared with other types of skin cancers, including squamous cell carcinomas and basal cell carcinomas 1,2. In the 1980s, IL-2 based cytokine therapy and adoptive T cells transfer therapy became available for melanoma patients but with poor responses and high toxicities 3,4. In the past decade, immune checkpoint blockade therapy (e.g. αPD-1, αPD-L1 and αCTLA-4) has revolutionized cancer treatment 5. However, the overall objective response rate for these anticancer immunotherapies still remains relatively low, ranging from 10-50% 6. Therefore, there is an urgent need to improve the efficacy of existing agents.

There is a well-documented epidemiological association of high body weight with increased risk of melanoma incidence and worse prognosis 7-9. Preclinical work with mouse B16 melanoma also showed a sex-dependent difference in αPD-L1 response 10. Specifically, B16 melanoma grown in males responded less robustly to αPD-L1 than that in females. Thus, the potential impact of obesity and sex warrants careful examination in the development of novel approaches to boost current anticancer immunotherapies.

We recently reported that adipose tissue, a major tumor microenvironment component, expresses high levels of PD-L1 during adipogenesis 11. Our published work also implicates adipocyte PD-L1 in modulating antitumor immunity and immune checkpoint blockade efficacy in breast cancer. In addition, we found that the PPARγ antagonist GW9662, known for its ability to inhibit adipogenesis, reduces adipocyte PD-L1 expression and boosts PD-1/PD-L1 blockade immunotherapy efficacy 11. In the current study, we used B16 mouse melanoma to examine the antitumor effect of the combination treatment of GW9662 and αPD-L1 immunotherapy. We also compared the therapeutic response in lean versus diet-induced obese mice in both sexes.

## Materials & Methods

### Mice

Wild type C57BL/6J male/female mice (Cat: #000664) and male C57BL/6J diet-induced obese (DIO) mice (Cat: #380050) were purchased from Jackson laboratory. For female DIO mice, regular C57BL/6J mice were fed high fat diet (HFD, Research Diets Inc. Cat: #D12492, 60 kcal% fat) or control diet (low-fat diet) (LFD, Research Diets Inc. Cat: #D12450B, 10 kcal% fat) starting from age of 6 weeks. For tumor studies in obesity and lean hosts, all mice were fed HFD/LFD for 12 weeks before tumor challenge. Oöphorectomy was done by bilateral removal of the ovaries under anesthesia. Oöphorectomized female mice were kept under special postoperative care for at least two weeks before further treatment. Oöphorectomized female at age of 8-10 weeks were used for the tumor studies. H&E staining was performed to verify complete ovary removal. Age-matched female, castrated and sham-treated male mice were purchased from Charles River Surgical Services at age of 20 weeks. Unless otherwise specified, 6 to 8-week-old mice were used. All animal procedures were carried out following the animal protocol approved by the Institutional Animal Care and Use Committee.

### Tumor challenge and treatments

5×10^5^ B16 melanoma cells were subcutaneously injected into mouse back flank per site of injection. Tumor volumes were assessed at indicated days post inoculation with calipers (0.5 × length × width^2^). Survival analysis was determined by animal death or tumor size ≥ 1000 mm^3^ or distress. αPD-L1 (BioXcell, Cat: #BE0101) and IgG2b (BioXcell, Cat: #BE0090) antibodies were administered at 10 mg/kg through intraperitoneal (i.p.) injection. Antibodies were administered twice per week. GW9662 (Sigma, Cat: #M6191) was dissolved in DMSO (25 mg/ml) and administered daily i.p. at 1 mg/kg in 150 μl PBS, starting from 14 days before tumor inoculation and sustaining throughout the entire experimental period.

### Flow Cytometry

Tumors were cut into small pieces, ground further by pestle in RPMI-1640 media, and passed through a 70 μm cell strainer to obtain a single-cell suspension. Cells were stained with viability Ghost Dye™ Violet 510 (Cat: #13-0870, Tonbo Biosciences) and washed with PBS. Cells were then blocked with αCD16/32 at a 1:100 dilution (Cat: #70-0161, Tonbo Biosciences). Surface staining was performed at 4^0^C for 30 min for CD45 (Super Bright 645, Cat: 64-0451-82, ThermoFisher), CD3 (violetFlour 450, Cat: 75-0032-U100, Tonbo Biosciences), CD4 (Brilliant Violet 605, Cat; 100548, BioLegend), CD8 (APC-Cy7, Cat: 557654, BD Pharmingen), CD44 (Brilliant Violet 785, Cat: 103041, BioLegend) and NK1.1 (PE/Cy7, Cat: 108714, BioLegend). For intranuclear staining, cells were permeabilized with a FoxP3/transcription factor staining kit (Cat: 00-5523-00, eBioscience) and stained for TCF-1 (PE, Cat: 564217, BD Bioscience). For intracellular cytokine staining, cells were incubated with αCD3/CD28 activation beads (Cat: 11452D, ThermoFisher) overnight at 37 °C, and subsequently in an activation cocktail with BD GolgiPlug™ (Cat: 550583, BD Biosciences) for 5 hr at 37 °C. Cells were permeabilized and stained for IFN-γ (PE-Cy7, Cat: 505826, BioLegend) and TNF-α (Alexa Fluor 647, Cat: 506314, BioLegend). Relevant controls include single color staining, fluorescence minus one (FMO), or isotype controls (BioLegend). Cells were assessed by BD FACSCelesta flow cytometer and analyzed with FlowJo and FACSDiva (BD Biosciences).

### Statistics

Unpaired student *t*-test was used to compare mean differences from two groups. One-way ANOVA followed by multiple comparisons was performed to compare mean differences from multiple groups. Two-way ANOVA was tested to compare tumor growth curves. Survival curves were examined by log-rank (Mantel-Cox) tests. All comparisons were performed using Graphpad Prism. *P*<0.05 was considered significant.

## Results

### GW9662 boosts αPD-L1 antitumor efficacy on melanoma in female mice

**Figure 1:**
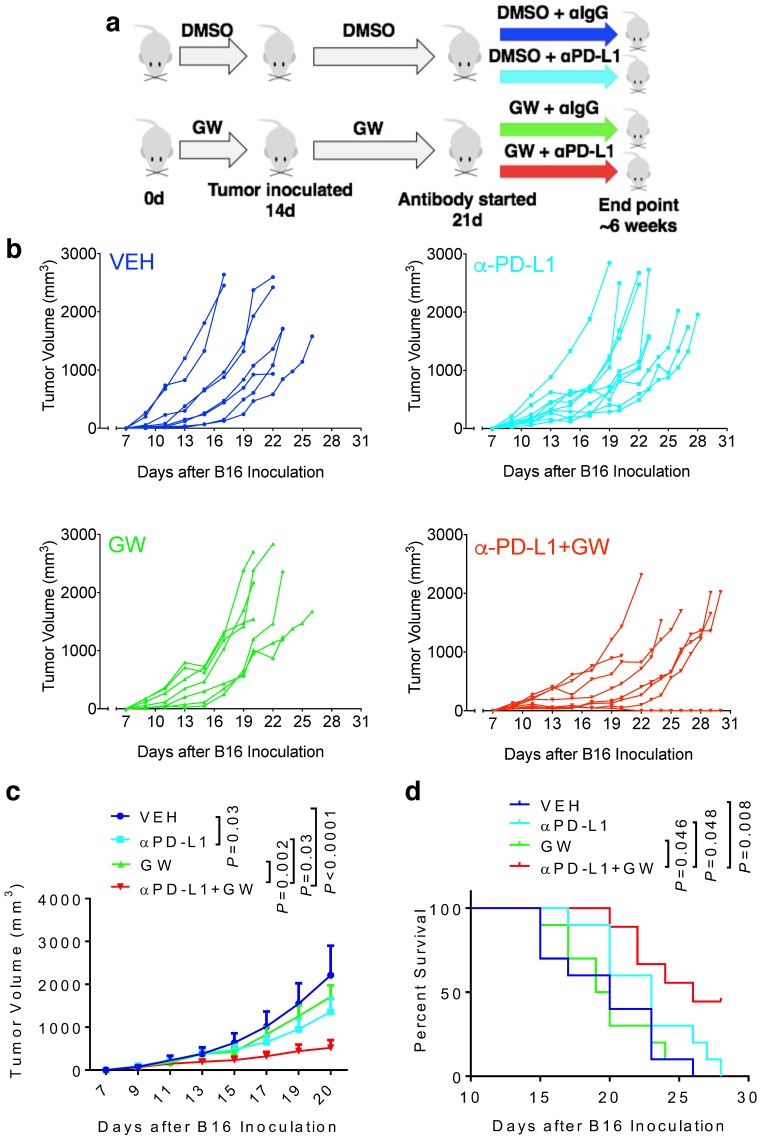
** GW9662 boosts αPD-L1 anti-melanoma efficacy in female mice. (A)** Scheme of treatment regimen for the four groups of mice. **(B)** B16 melanoma tumor growth curves from individual mice with four-arm treatments. Average tumor volume **(C)** and **(D)** survival curves in female mice with four-arm treatments, VEH: DMSO+αIgG (n=8). αPD-L1: DMSO+αPD-L1 (n=10). GW: GW9662+ αIgG (n=7). αPD-L1+GW: αPD-L1+GW9662 (n=8). *P* values as indicated.

To determine whether melanoma responds to GW9662 alone and in combination with αPD-L1, we first pre-treated C57BL/6 female mice with either GW9662 or DMSO vehicle for two weeks and then challenged them with B16 melanoma cells. Mice in the DMSO- and GW9662-treated groups were further divided into the following four treatment groups with either isotype IgG control or αPD-L1: (1) vehicle only (with isotype control), (2) αPD-L1, (3) GW9662, and (4) αPD-L1+GW9662. All treatments in these four groups lasted for the duration of the experiment (Fig. 1a). Mice treated with αPD-L1 alone exhibited significant reduction in tumor volume (Fig. 1b-1c) without obvious improvement in overall survival (Fig. 1d). GW9662 treatment alone had no effect on tumor volume or survival (Fig. 1b-1d). In contrast, combination treatment with αPD-L1 plus GW9662 significantly slowed tumor growth (Fig. 1b-1c) and prolonged survival (Fig. 1d) compared to either vehicle or single treatment groups. We conclude that GW9662 can boost therapeutic efficacy of αPD-L1 immunotherapy against B16 melanoma in females. This finding corroborates and extends our previous results in murine mammary tumor models 11.

**Figure 2:**
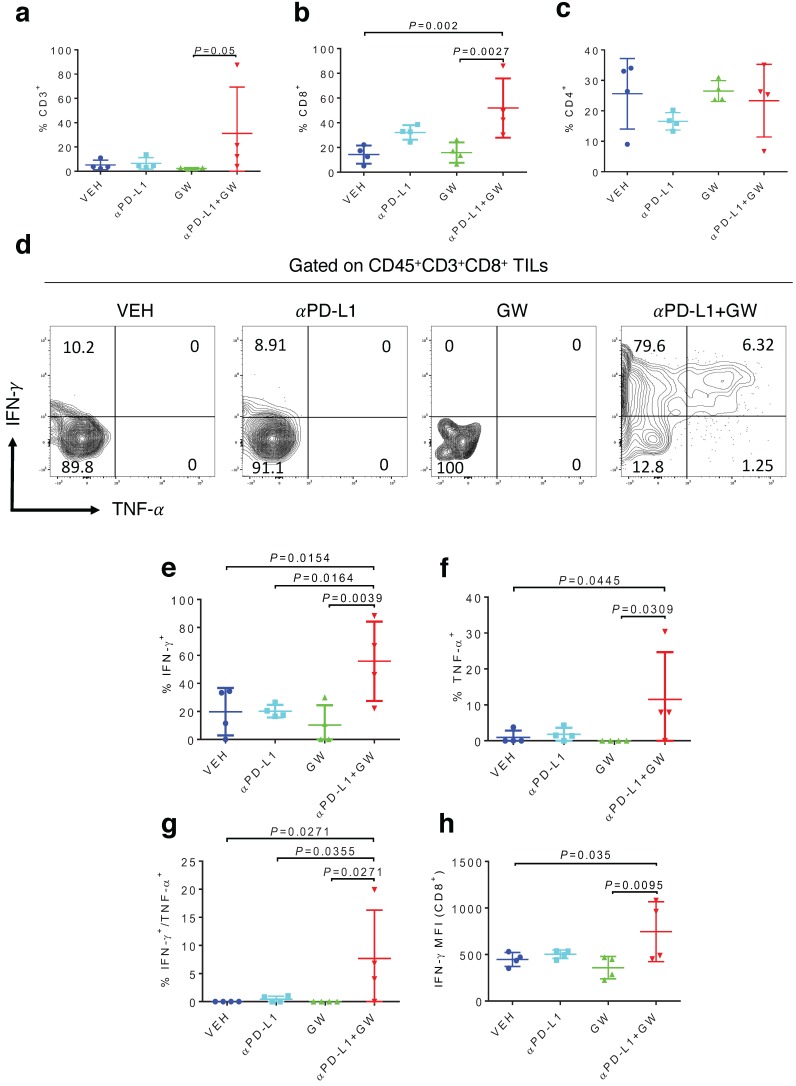
** Combination of GW9662 and αPD-L1 elicits more tumor-infiltrating T cells and anti-tumor cytokines in CD8^+^ TILs versus single agents. (A)** Percentage of CD3^+^ T lymphocytes of live CD45^+^ cells from tumors isolated 13 days post tumor injection in female mice following various treatments as indicated. **(B)** Percentage of CD8^+^ T cells percentage (of CD45^+^CD3^+^ cells) in αPD-L1 and GW9662 combination treatment group. **(C)** Percentage of CD4^+^ T cells (of CD45^+^CD3^+^ cells). **(D)** Representative flow cytometry of the IFN-γ and TNF-α staining in CD45^+^CD3^+^CD8^+^ TILs. **(E)** IFN-γ^+^, (f) TNF-α^+^ and **(G)** dual positive IFN-γ^+^ TNF-α^+^ percentage gated on CD45^+^CD3^+^CD8^+^ T cells. **(H)** Mean fluorescent intensity (MFI) of IFN-γ, an indicator of cytokine production per cell in various treatment groups. N=4 mice per group. VEH: DMSO+αIgG/. αPD-L1: DMSO+αPD-L1. GW: GW9662+αIgG. αPD-L1+GW: αPD-L1+GW9662. Data represent mean ± SD. *P* values as indicated.

To characterize immune effects of the combination of αPD-L1 and GW9662, we analyzed tumor infiltrating lymphocytes (TILs). Notably, percentages of total CD3^+^ T cells (Fig. 2a), CD8^+^ T cells (Fig. 2b) and CD3^-^NK1.1^+^ natural killer cells (Fig. S1a) were significantly elevated in the combination group versus single-agent and control groups. CD44^+^ activated CD3^+^ T cells percentage was also significantly increased after combination treatment (Fig. S1b). The effect of GW9662/αPD-L1 combination on CD8^+^ cell prevalence was substantially higher than that on CD4^+^ T cells (compare Fig. 2b and 2c). However, in both CD8^+^ and CD4^+^ T cells, percentages of cells double positive for IFN-γ and TNF-α (Fig. 2d-2g for CD8^+^ cells, Fig S2a-2d for CD4^+^ cells) and IFN-γ^+^ mean fluorescence intensities (MFI, Fig. 2h, Fig S2e) were significantly higher in the combination group versus control and the majority of the single treatment groups. Collectively, these immune data are consistent with the concept that combination treatment of αPD-L1 and GW9662 elicits enhanced antitumor effects versus single agents, and are consistent with data that we previously published in mouse mammary tumor models 11.

### Male hosts are refractory to GW9662/αPD-L1 combination treatment in a castration-independent manner

**Figure 3:**
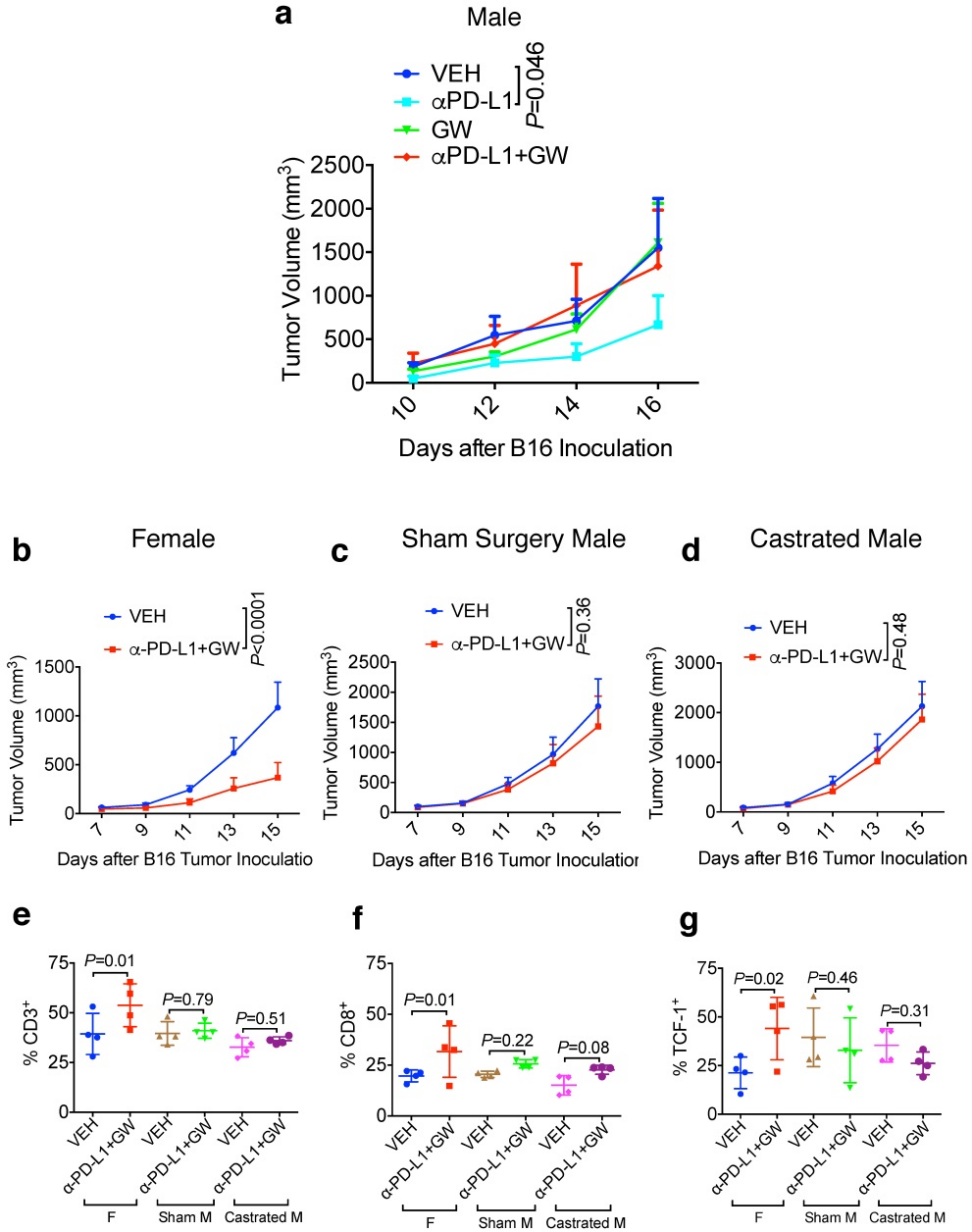
** Male hosts are refractory to the GW9662 + αPD-L1 combination treatment in a castration-independent manner. (A)** B16 tumor growth curves in normal male mice (n=5 per group). **(B-D)** B16 tumor growth in age-matched female (n=10 per group) (b), sham surgery-treated male (n=9 for VEH, n=8 for αPD-L1+GW) (c) and castrated male mice (n=9 for VEH, n=10 for αPD-L1+GW) (d). VEH: DMSO+ αIgG. αPD-L1: DMSO+αPD-L1. GW: GW9662+αIgG. αPD-L1+GW: αPD-L1+GW9662. **(E-G)** TIL analysis for B16 tumors in age-matched female, sham-treated male and castrated male mice. Percentage of (e) CD3^+^ (of CD45^+^), (f) CD8^+^ (of CD45^+^CD3^+^), (g) T cell stem cell marker TCF-1^+^ (of CD45^+^CD3^+^CD8^+^). N=4 mice per group for panel e-g. **(F)** Female. Sham M: sham surgery-treated male. Castrated M: castrated male. Data represent mean ± SD. *P* values as indicated.

Male melanoma patients tend to have a worse prognosis and develop more aggressive tumors versus females 10,12. We therefore determined whether the combination treatment of GW9662 plus αPD-L1 exerted a similar tumor-inhibiting effect on B16-bearing male mice. Consistent with the data in females, αPD-L1 alone effectively suppressed tumor growth in male hosts (Fig. 3a); however, in contrast to the antitumor effect of combination in female mice, the combination treatment did not significantly reduce tumor growth compared with vehicle alone in males. Strikingly, GW9662 abrogated the effect of αPD-L1 as a single agent in males (Fig. 3a). These results indicate that there is a sexually dimorphic action of GW9662 combined with αPD-L1.

To determine the contribution of male sex hormones to the observed sexual dimorphism, we compared the effect of the GW9662 + αPD-L1 combination treatment on melanoma tumor growth in age-matched females, sham surgery-treated males, and surgically castrated males. In this experiment, we used approximately 20-week-old mice to mimic a typical human melanoma patient population. While female mice again experienced significant tumor growth suppression by combination treatment (Fig. 3b), castration was unable to restore the response to the combination treatment in male mice (compare Fig. 3c and 3d). By flow cytometry, we found that combination treatment of female mice significantly increased CD3^+^ and CD8^+^ T cell tumor infiltration as well as CD8^+^ cells expressing the T cell stem cell transcription factor TCF-1, but the same treatment failed to exert similar beneficial effects on either sham-surgery treated or castrated males (Fig. 3e-3g). These results indicate that male sex hormones are unlikely responsible for recalcitrance of male mice to GW9662 + αPD-L1 combination treatment, or the ability of GW9662 to reduce αPD-L1 efficacy. In a reciprocal tumor study using oöphorectomized females, we found that tumor response to the combination treatment remained intact in female hosts after ovary removal (Fig. S3a-3c), indicating that therapeutic effects of the GW9662 + αPD-L1 combination on B16 melanoma growth are likely independent of female sex hormones.

### Diet-induced obesity abrogates aPD-L1 effect in male and attenuates aPD-L1 + GW9662 combination antitumor efficacy in female

**Figure 4:**
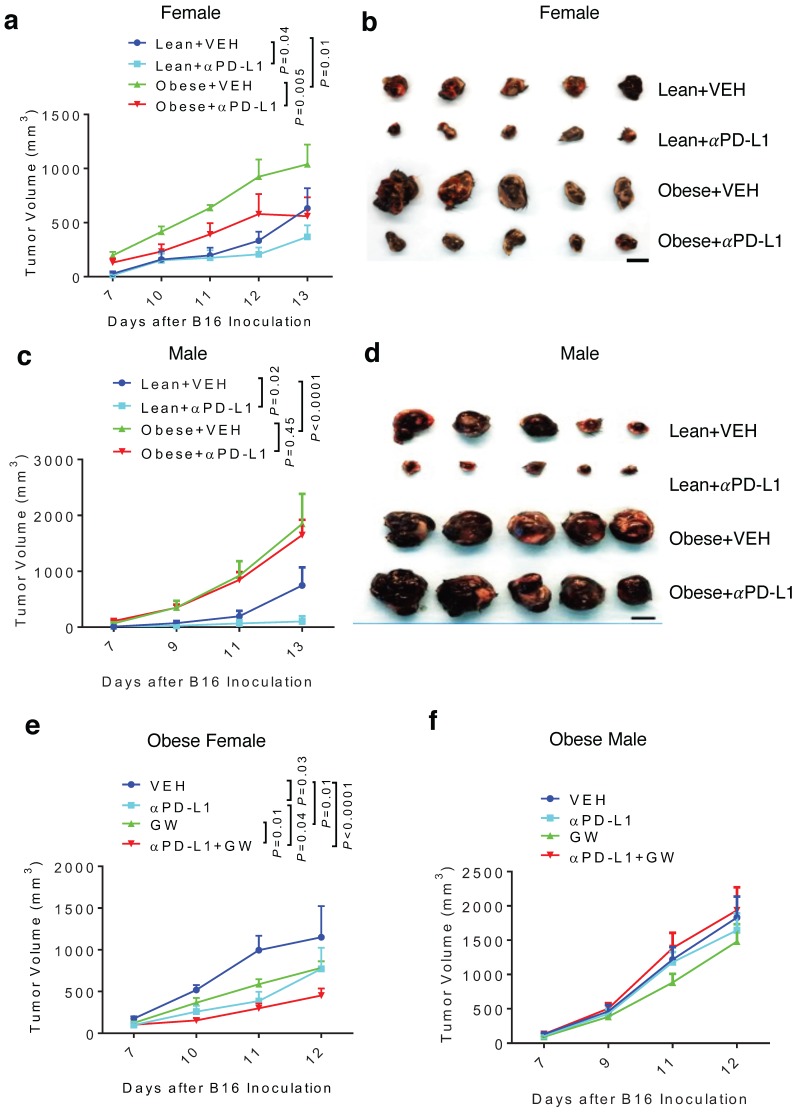
** Diet-induced obesity abrogates αPD-L1 effect in males and attenuates αPD-L1 + GW9662 combination antitumor efficacy in females**. **(A)** Tumor volume (n=8 for lean + VEH, n=7 for lean + αPD-L1, n=4 for obese + VEH, n=5 for obese + αPD-L1) and **(B)** tumor size of B16 melanomas in lean/obese female mice. **(C)**Tumor volume (n=9 for VEH groups, n=10 for αPD-L1 groups) and **(D)** tumor size in male mice. Tumor volume of **(E)** obese female (n=5 for VEH, n=5 for αPD-L1, n=10 for GW, n=8 for αPD-L1+GW) or **(F)** obese male mice (n=10 per group). VEH: DMSO+αIgG. αPD-L1: DMSO+αPD-L1. GW: GW9662+ αIgG. αPD-L1+GW: αPD-L1+GW9662. Scale bar: 1 cm. Data represent mean ± SEM. *P* value as indicated.

Because obesity is associated with more aggressive melanoma 8, we tested antitumor effects of GW9662 and αPD-L1 in obese mice. Females and males were fed either high-fat or control chow for 3 months. As expected, mice fed the high-fat diet gained significant weight compared to mice fed the control diet (Fig. S4a-4b). Obese and lean mice of each sex were challenged with B16 melanoma cells, followed by treatment with αPD-L1 or isotype IgG control. Consistent with the previously reported correlation between obesity and more aggressive tumor growth 8, control-treated tumors in the obese female and male groups grew faster versus in corresponding lean groups (Fig. 4a-4d, Fig. S4c-4f). Treatment with αPD-L1 significantly reduced tumor growth in obese female mice (Fig. 4a-4b, Fig. S4c-4d). In contrast, tumors in obese males were completely refractory to αPD-L1 treatment (Fig. 4c-4d, Fig. S4e-4f). Further, tumor immuno-phenotyping shows that αPD-L1 significantly increases CD45^+^ total leukocytes and CD3^+^ T cells infiltration in lean but not obese male hosts (Fig. S5a-5b).

Next, we asked whether GW9662 could boost αPD-L1 efficacy in obese mice. In obese female mice, the GW9662 + αPD-L1 combination treatment still achieved greater tumor inhibition than the single-agent treatment (Fig. 4e), although the added effect of the combination treatment was less pronounced than that in lean females (Fig. 1a-1b). However, obese males did not respond to either single or combination treatment (Fig. 4f).

## Discussion

In our current syngeneic mouse melanoma study, the combination treatment of GW9662 and αPD-L1 antibody exhibits superiority over single-agent treatment in lean and obese female mice, but not their male counterparts, suggesting a confounding factor(s) that modulates the GW9662 action. Our finding in the preclinical model is reminiscent of previously documented clinical observation that female sex is an independent positive predictor of favorable outcome for melanoma patients at all stages 13-15. The underlying mechanism could be complex and multifactorial. While sex hormones have been implicated in various immune responses 16, there are numerous reports of sex hormone-independent sexual dimorphisms caused by fundamental genetic differences in sex chromosomes. Possible underlying mechanisms include X chromosome-related gene dosage effects, X chromosome inactivation and epigenetic modifications 17. For example, X chromosome linked, sex-determining region Y box 9 (SOX9) transcriptionally regulates CEACAM1 expression in melanoma cells and thereby their immune resistance 18. SOX9 was also found to be able to regulate interleukin 8 production in human dental pulp cells in response to inflammatory cues such as TNFα 19. Aneuploidy studies found that males expressing an excess X chromosome have a higher chance of developing autoimmunity such as systemic lupus erythematosus 20. A genome-wide study in mice also found an inverse correlation between Y chromosome-specific gene copy number variation and immune cell-specific gene upregulation 21. These studies suggest sex chromosome-dependent and independent sexually dimorphic immune responses in various physiological and pathological contexts. Thus, the complex effects of sex on tumor growth and immunotherapy response could be immune cell-, organ-, and/or tumor-specific. These important questions require additional investigation.

PPARγ is known to exert profound effects on both tumor cells and host immune cells. PPARγ is expressed in a wide range of immune cells, including T helper cells, monocytes and regulatory T cells. It has been shown that PPARγ is significantly upregulated in activated macrophages and suppresses macrophage cytokine secretion 22. PPARγ is also found to suppress helper T cell proliferative response and IL2 secretion 23. Furthermore, studies also found that PPARγ is critical for mediating regulatory T cell accumulation and inhibitory function 24,25. Of note, some of the immune cell types that are regulated by PPARγ exhibit a distinct sex prevalence 26,27, which could contribute to the sexually dimorphic response to PPARγ antagonist observed in our current study.

The blunting effect of obesity on GW9662 and αPD-L1 could be due to obesity-triggered elevation of adipocyte-derived factors such as PD-L1, leptin, and IL-8, all of which are known to antagonize host antitumor immunity and the therapeutic efficacy of checkpoint blockade immunotherapies 11, 28-30. Males are known to be more susceptible to develop HFD-induced obesity than females 31. The obesity-associated body weight increase is largely due to the increase of fat tissue, which mainly consists of adipocytes. Therefore, the larger body weight of obese males versus their female counterparts could account for the exacerbated dampening of antitumor immune response through prevention of αPD-L1 antibody from reaching to their targeted cell populations within the tumor microenvironment. In addition, distinct actions of sex chromosome-related biological factors as described above could further contribute to the differential effect of diet-induced obesity on therapeutic response in male and female hosts.

We showed previously that adipocyte PD-L1 can suppress T cell activation and the immune-boosting effect of αPD-L1 on T cells *in vitro* 11. Also, we demonstrated that GW9662 can downregulate PD-L1 expression both *in vitro* and *in vivo*. In the melanoma model used in the current work, GW9662 likely boosts tumor-infiltrating T cell activation and response to αPD-L1 through a similar mechanism. Besides breast cancer and melanoma, adipocytes are also enriched in tumor microenvironment of other cancer types including ovarian, colorectal, pancreatic and prostate cancer. Specifically, pancreatic adipocyte infiltration has been positively associated with pancreatic ductal adenocarcinoma in humans 32. In addition, pancreatic tumor-surrounding adipocytes have been shown to promote tumor progression through modulating the pancreatic tissue fibrosis and inflammatory milieu 33. Furthermore, adipocyte-enriched microenvironments also serve as a primary fuel of tumor metastasis 34. For example, omental fat is a primary site for ovarian cancer cell migration and is known to facilitate tumor malignant outgrowth. We surmise that the combination strategy of targeting adipocyte and immune checkpoint could yield better therapeutic response of other adipocyte-enriched tumor types.

A recent meta-analysis of patients treated with metastatic melanoma suggests that obese melanoma patients respond better to immune checkpoint blockade immunotherapies, but not dacarbazine or targeted small molecules 35. Furthermore, compared to patients with normal body mass index, obese patients had improved progression-free survival, but not overall survival in male patients treated with αPD-1 or αPD-L1. However, it is worth noting that most obese cancer patients involved in this clinical trial were treated for metabolic syndrome-related comorbidities. Because several antidiabetic drugs have potential antitumor effects; either by themselves or in combination with immunotherapy in preclinical melanoma models 36,37, they could confound interpretation of obesity association with immunotherapy outcomes. In contrast to the afore-mentioned findings, a separate cohort study of melanoma patients reported that obese and overweight patients receiving αPD-1 tend to have worse progression free survival for patients compared with normal weight patients 38. Furthermore, a recent retrospective analysis of cancer patients receiving αPD-1/PD-L1 therapies found that obese patients with higher BMI (≥30) tend to have shorter median time to treatment failure compared with patients with lower BMI (25-30) (7.3 months vs. 10.3 months) 39. Yet in another recently published preclinical study, αPD-1 was shown to work better in melanoma-bearing obese mice 40, which is in contrast to our finding of obesity-related therapeutic resistance to αPD-L1, especially in male mice. One possible explanation is that, in addition to the PD-L1/PD-1 interaction, PD-L1 and PD-1 also engage CD80 and PD-L2, respectively, which could lead to distinct αPD-L1 and αPD-1 effects, especially if these additional protein partners of PD-L1/PD-1 are differentially affected by obesity as is known for PD-1 and PD-L1 41,42. Collectively, these conflicting findings highlight the complex relationship between immune checkpoint blockade efficacy and obesity and therefore warrant further investigations.

In summary, our current study uses syngeneic melanoma mouse models to show that pharmacologic inhibition of PPARγ boosts anticancer immunotherapies in a sexually dimorphic and obesity-dependent manner. Furthermore, we provide evidence that the lack of response in male mice is sex hormone-independent and that diet-induced obesity can blunt antitumor efficacy of combination treatment with the PPAR-γ antagonist and αPD-L1. Taken together, our data point to the relevance of gender and adiposity to the efficacy of specific immune checkpoint blockade agents in the clinical settings, and provide important leads for investigation in addition to other recent advances in understanding obesity effects on melanoma immunotherapy. Of great relevance is to understand if these effects are particular to melanoma or extend to additional cancers.

## Supplementary Material

Supplementary figures.

## Abbreviations

GW: GW9662
TILs: Tumor infiltrating lymphocytes
DIO: diet-induced obese
i.p.: intraperitoneal
VEH: vehicle
αPD-L1: anti-programmed death-ligand 1
αPD-1: anti-programmed cell death protein 1
PPARγ: Peroxisome proliferator-activated receptor gamma
