# Supplementary material for: PPARγ inhibition boosts efficacy of PD-L1 Checkpoint Blockade Immunotherapy against Murine Melanoma in a sexually dimorphic manner
Source: Int J Biol Sci. 2020 Mar 5;16(9):1526–35. doi: 10.7150/ijbs.42966 (PMC7097912; doi:10.7150/ijbs.42966)

Supplemental Fig. 1 GW9662 + αPD-L1 combination treatment increases TIL infiltration and activation in female mice

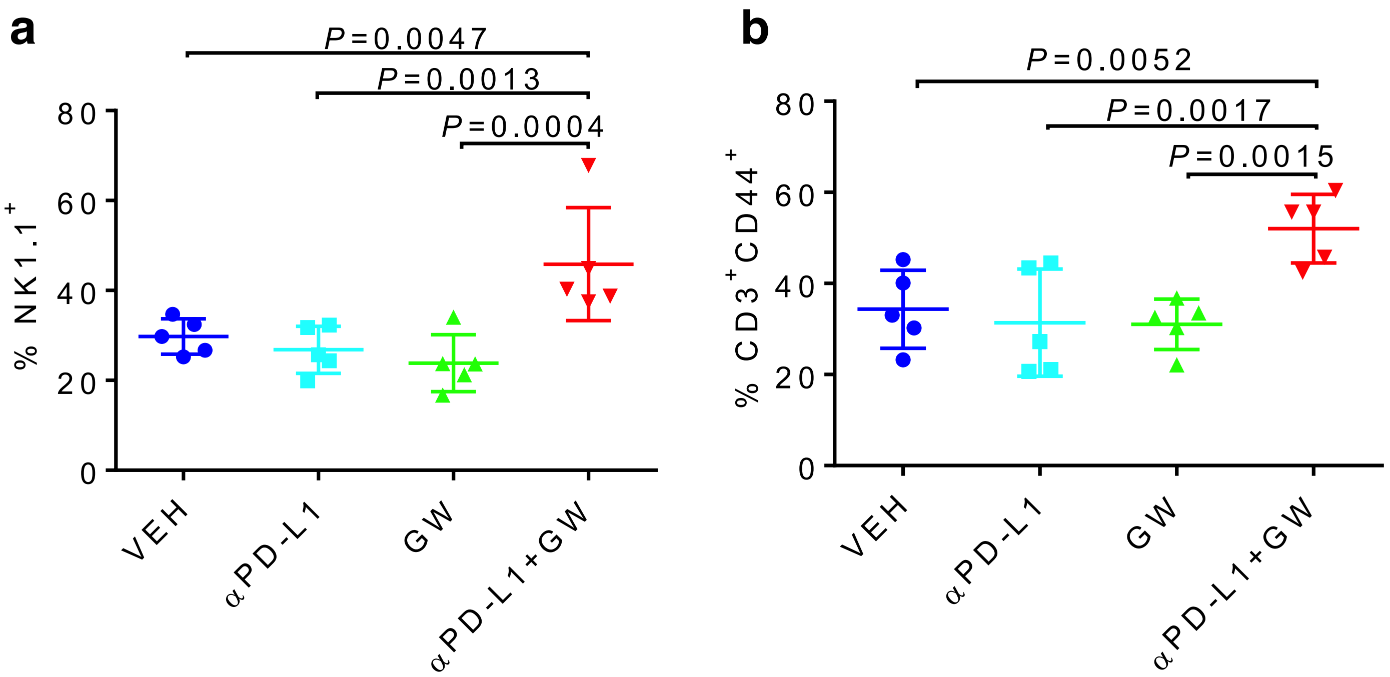

Supplemental Fig. 2 GW9662 + αPD-L1 combination treatment enhances antitumor immunity in CD4+ TILs in female hosts

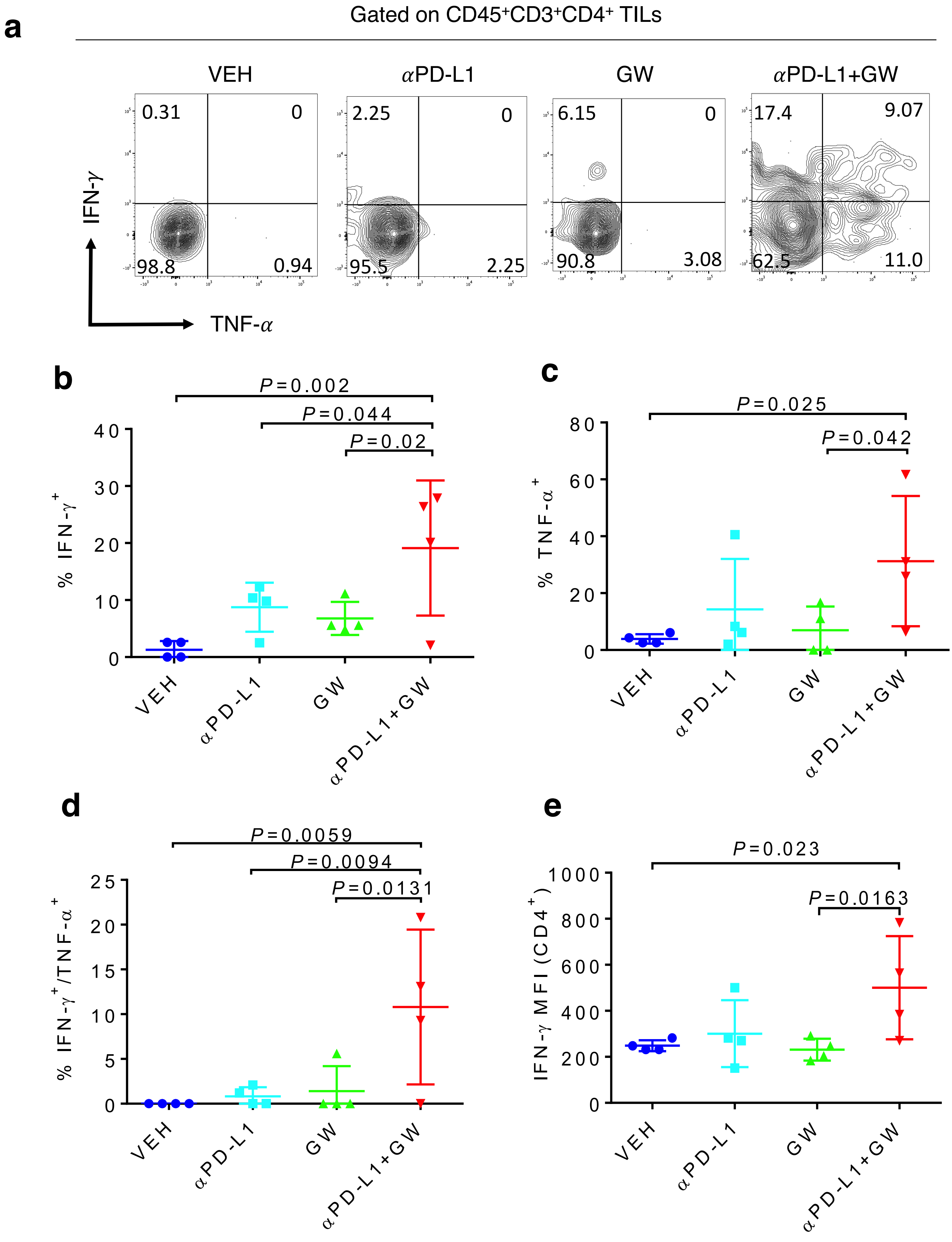

Supplemental Fig. 3 Ovaries are dispensable for antitumor efficacy of αPD-L1+ GW9662 treatment

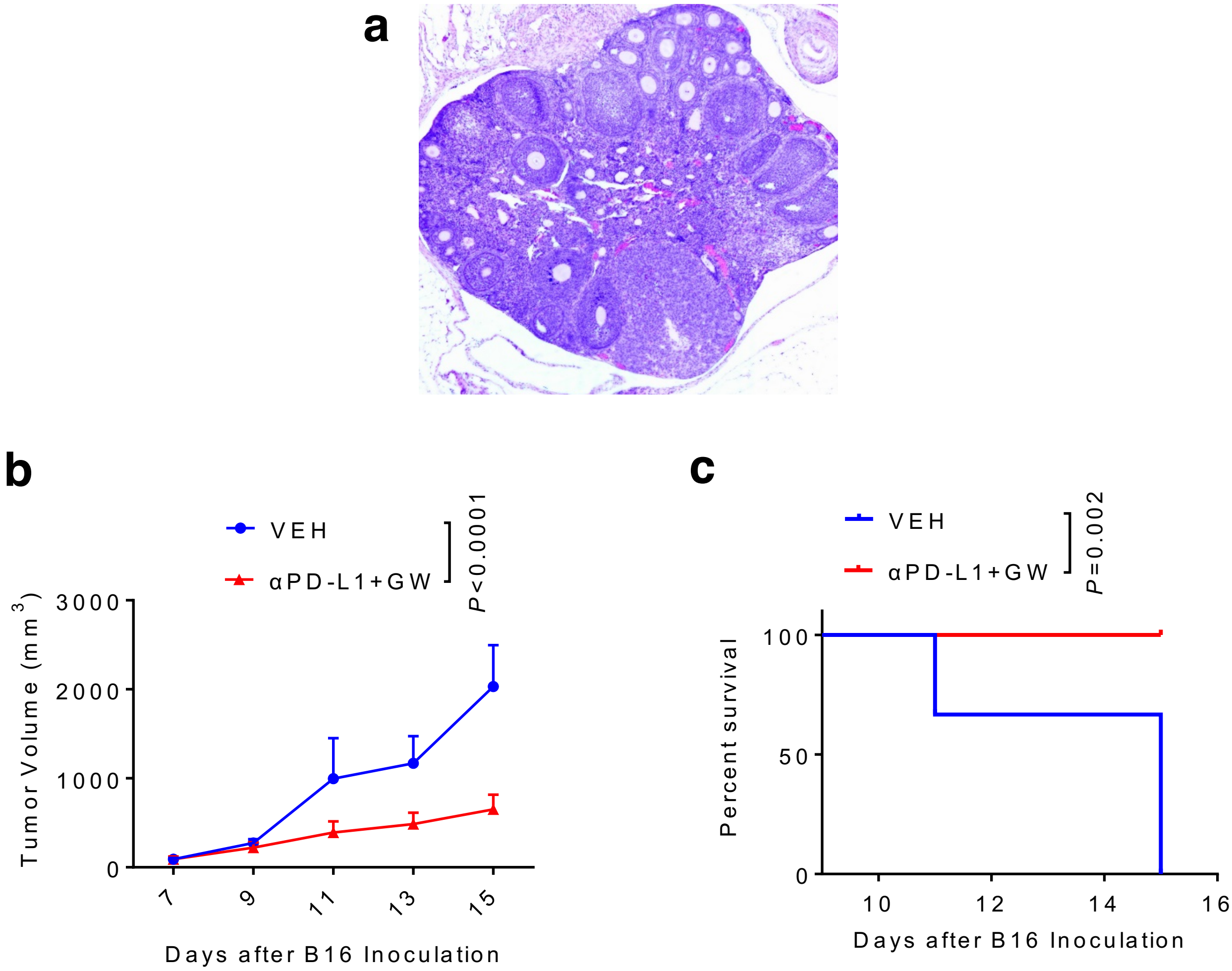

Supplemental Fig. 4 High-fat diet effect on B16 melanoma response to αPD-L1 in male and female mice

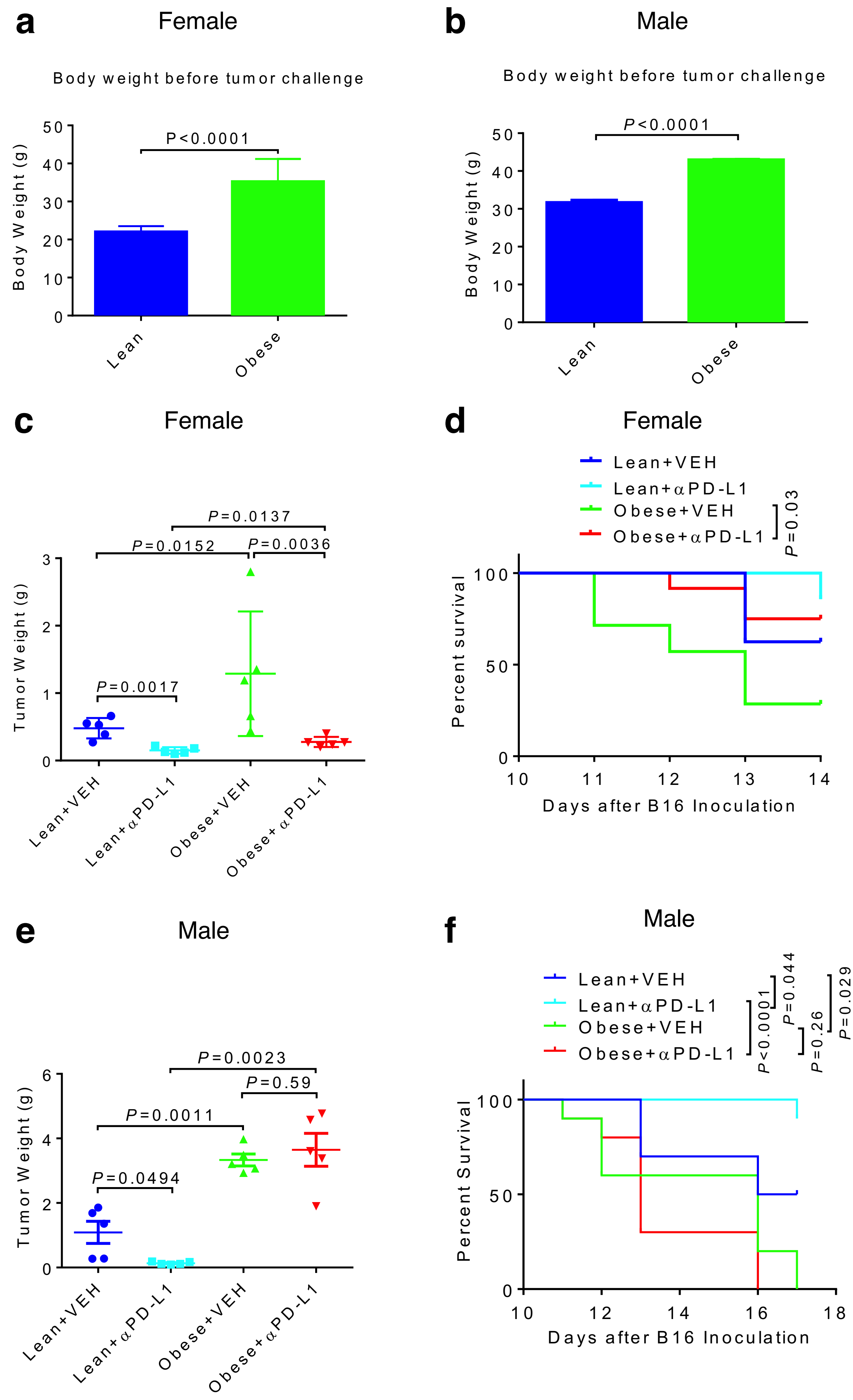

Supplemental Fig. 5 Immuno-phenotyping of B16 tumor-bearing lean and obese male mice

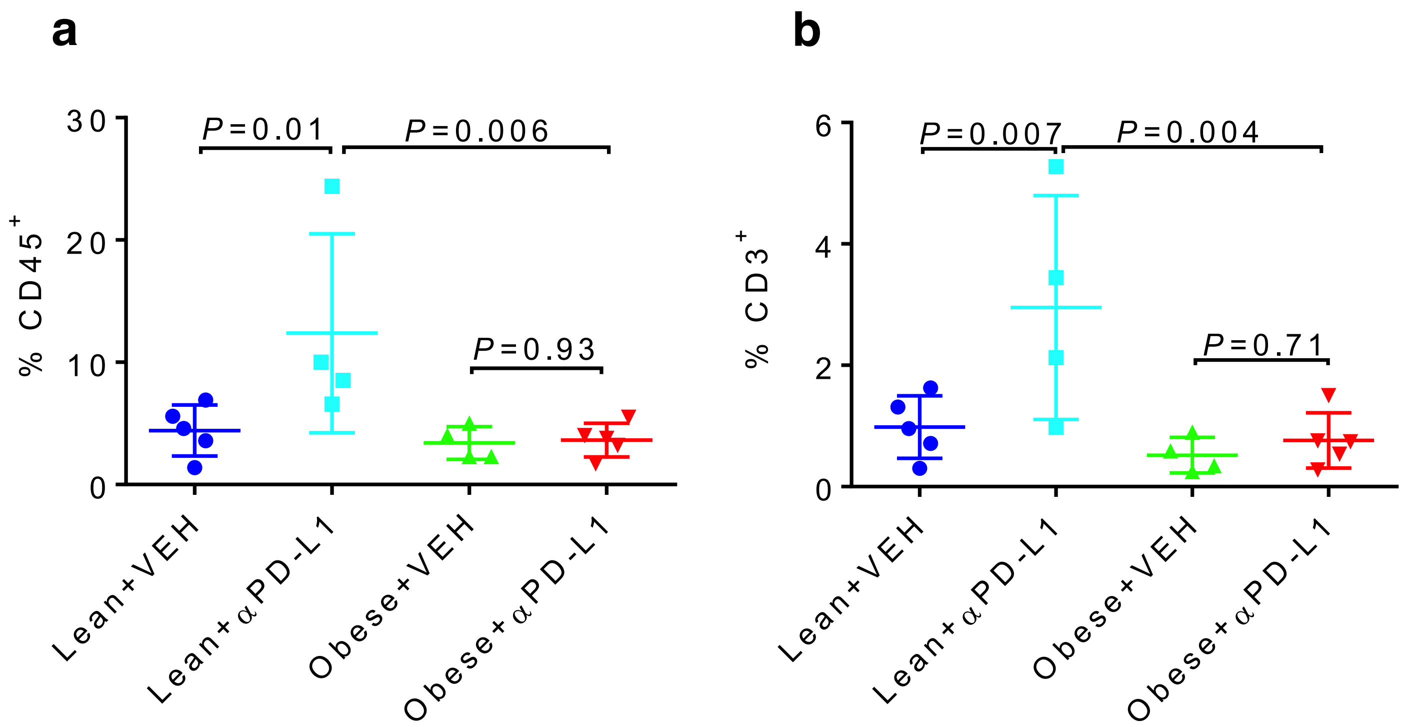

Supplement: Supplementary file 1 — Supplementary figures. [file ijbsv16p1526s1.pdf]
